# Progress in Psychiatry

**Published:** 1902-06-21

**Authors:** 


### PROGRESS IN PSYCHIATRY.

(*Continued from page* 153.)

**The Insanity of Adolescence.**—The mental disorders of adolescence occur in both sexes after the age of 17 or 18 years, and seriously disturb the moral and sexual life while they obscure or blunt the intelligence. This is shown by the prevalence of morbid impulses and instincts rather than by the evolutions of delusions. Indeed, delusions of slow, gradual, and systematic evolution, such as are characteristic of paranoia, do not characterise the insanity of adolescence. The insanities of adolescence have certain common features running through them all which serve to mark them as a class by themselves. Hence they have been grouped together, as the result of recent researches,[6] under the title dementia præcox, or the precocious dementia of adolescents. Both Pinel and Esquirol regarded this affection as a form of idiocy, and it was reserved for Morel, of Belgium, in the middle of the nineteenth century, to show its true place in the scheme of mental diseases. Dementia præcox is not a motley group of various vesanic or affective insanities,[7] but a special malady characterised by mental enfeeblement, progressive in character, supervening as a rule in adolescence and terminating most often in a moderate or marked degree of dementia. This termination is common to all its forms. The initial disturbances are those of the moral and sexual functions represented in the brain. A co-existent defect of volition intelligent attention and self-control is manifested about the same time or soon afterwards. When the disease has nearly run its course the subsidence of acute manifestations brings into relief a notable degree of mental enfeeblement which is present and which remains thereafter a striking feature of the disease. Christian, of Charenton, states[8] that this affection is much more frequent than is usually supposed, and that he has met with 100 cases during 19 years. The characteristic symptoms are summed up by him as follows: (*a*) its appearance at the period of puberty and adolescence (between the ages of 15 and 25); (*b*) the variable and fleeting delusions at the onset and in the early stages of the disease (the delusions being of a persecutory nature, *e.g.*, as regards unseen enemies, robbers, scandal-mongers, poisoning of the patient's food, the devil and hell being visible, or erotic and obscene insults being sustained); (*c*) the frequent occurrence of sudden impulsive acts or short sharp outbreaks of violence; and (*d*) the rapid lapsing of the acute symptoms into a condition of mental weakness more or less pronounced. The period of incubation of the disease is described by him as one of mental or cerebral fatigue with inaptitude for work. Then follows the period of impulses, excitement, confusion, or delusions, which constitute the acute manifestations of the disease. Among the causes mentioned by the same author are, first and chiefly, debilitating causes and especially cerebral over-exertion (*surmenage*); secondly, a hereditary neurotic or insane taint inherited from the parents, the parental or ancestral vice being one which according to Bevan Lewis[9] has shown itself late in the life of the parent; and thirdly, the disturbing effects of puberty and adolescence on the brain. As regards sex, Christian states that the disease is more frequent in the male than in the female, but that the disparity is gradually growing less. Bevan Lewis states that 40 per cent. of the patients had an insane heritage, 10·6 per cent. afforded a history of ancestral epilepsy, and 9·3 per cent. a history of apoplectic seizures in the parents. As regards age it appeared, according to the same author, that out of 77 cases, occurring at all ages from 12 to 21 years inclusive, 56, or nearly three-fourths of the total, were from 18 to 21 years of age. There were only three cases up to the age of 15 years, and nine cases each during the sixteenth and seventeenth years. Of 13 cases recorded with detailed clinical histories by Masselon,[10] the ages were as follows. In five males the ages varied from 16 to 23 years of age, and in three single females the ages were respectively 17, 17, and 21 years. In married women the onset of the disease appeared later, the ages varying from 26 to 35 in five cases recorded by the same author. Kræpelin in his "Psychiatrie" gives a diagram showing that more than 60 per cent. of the cases occurred before the twenty-fifth year, and Trommer, of Hamburg, in a monograph on dementia præcox shows that a small proportion of cases occurred between the ages of 25 and 35 years, but that the disease was essentially one of the period of puberty and adolescence. The clinical varieties of dementia præcox are as follows:—

(*a*) Simple, non-delusional form. This is characterised by a mild degree of apathy and slow and progressive mental enfeeblement.

(*b*) Delusional or hebephrenic form. This is characterised by a medley of mental states, such as maniacal excitement, depression of spirits, hallucinations (most visual, and less frequently auditory), and mental confusion. The hallucinations and delusions are of mobile and fleeting character, and the mood and temper of the patient are highly variable. The depression of spirits which shows itself is not a true melancholia, but a shallow depression varied by periods of liveliness, gaiety, and silly acting and chattering. Showy and histrionic attitudes and demonstrations are indulged in by the patient, mixed with grimaces and laughter without cause. Exhortations after the manner of an orator, often with incomprehensible words and sentences, are indulged in. Under the influence of hallucinations and delusions the patient is liable to give way to dangerous transitory outbreaks of violence, or to perpetrate petty crimes if he be free and at large, or to wander about and act strangely and to exhibit symptoms of marked mental confusion and silliness when questioned. This is the commonest variety of the disease.

(*c*) Catatonic form. This variety of dementia præcox is marked by phases of stupor, of passive resistance to everything which is required to be done, of cataleptic attitudes in which the limbs remain for long periods in extraordinary positions, of mutism, and of verbigeration—the latter phenomenon consisting of the utterance and repetition of meaningless sounds and syllables—and of occasional, sudden, and unforeseen impulses to violence. The classical picture of this form of insanity was drawn by Kahlbaum more than 20 years ago. He has described "the bent body, the stiffened arms and legs, the wrinkled brow and downcast eyes, the stubborn silence of negativism . . . the wasting, the vaso-motor disturbances, the stereotyped way of doing things, the verbigeration, the isolated convulsions, and the unforeseen and sudden impulses to violence" which the patient exhibits. The psycho-pathology of this peculiar morbid condition has been ably described by Brosius. The psychosis is not, as Kahlbaum supposed, a simple mania; the quick flow and change of ideas, and the lively and whimsical mood of mania proper being absent in catatonic dementia præcox. The psychical process in catatonia appears on the contrary as a monotonous flow of words and phrases, often repeated, the manner is dramatic or pathetic, and bodily restlessness is not marked. Kahlbaum remarked that catatonic patients were generally of a "soft temperament, quiet and gentle," which is true. In several cases quoted and described by Pickett[11] persecutory delusions were common. "A persecutory mood," says the author, "so to speak, was always present."

(*d*) The paranoid form. This form is marked by rapid intellectual enfeeblement with sensorial disturbances and delusional conceptions evolved in an atmosphere of apparent mental lucidity. This is a comparatively rare form.

(*e*) The criminal form is, like the former, comparatively rare, as compared with the other forms of dementia præcox. It manifests itself by the development of strong criminal instincts, especially towards homicide, theft, and arson. The patient manifests these symptoms periodically, the impulses appearing to be potent and irresistible at such times. It is an extreme pathological development of a psychosis, which in its milder form is exemplified by the hooligan. Patients of this class are not to be confounded with the moral imbecile whose anti-social or criminal instincts arise from congenital defect.

The terminal states of dementia præcox possess a curious similarity. After the occurrence of one or two acute attacks (or in patients with marked heredity several relapses and rapid recoveries) a permanent degree of mental enfeeblement is observed, though the progress of mental decay is variable. "Certain hebephreniacs remain on the borders of imbecility,"[12] others fall almost from the beginning into a condition of profound apathy or stupidity. Among the bodily symptoms observed in dementia præcox are exaggeration of the knee-jerks, dilatation and sluggishness of reaction of the pupils, cardiac and vaso-motor feebleness (coldness and blueness of the extremities owing to feeble or slow circulation), secretory troubles, and occasionally fits of a hysterical or convulsive character. As regards curability Masselon states, from a careful study of statistics collected from various sources, that 8 per cent. of patients recover from the hebephrenic and 13 per cent. from the catatonic form of adolescent insanity.[13] The presence of marked anæmia, the occurrence of obstinate constipation, and vicious indulgence in masturbation are symptoms frequently associated with the psychoses of adolescence. Excessive masturbation is of itself prone to cause dulness and apathy with depression of spirits, timidity, loss of energy and of irritative inaction—in short, cerebral neurasthenia. This condition is more potent for harm in the male sex than in the female, and in subjects with neurotic predisposition it may be the exciting cause of adolescent insanity in its simple or hebephrenic forms. Treatment should be directed to the cure of such vicious habits when present. The application of cold baths (spinal douche and shower- or hip-baths), massage, and out-door exercise will be beneficial to such cases. Anæmia, when present, should be corrected by suitable hæmatinics and laxatives (iron, aloes, Easton syrup). Sedatives should be given for insomnia. Full nourishment should be given where there is emaciation, and supervision is necessary to guard against dangerous impulses.

[6] Same references as 4. [7] Sérieux, Gazette Hebdomaire, April 10, 1901. [8] Annales Medico-Psychologiques, 1899. [9] Textbook of Mental Diseases, 1899. [10] Thèse de Paris, 1902. [11] Journal of Nervous and Mental Disease, Aug. 1901. [12] Christian, Annales Medico-Psychologiques, 1899. [13] Same reference as 10

